# Lifting Efficiency of Barbed Sutures for Potential Face Lifting Application: A Parametric Analysis

**DOI:** 10.1111/jocd.70730

**Published:** 2026-03-06

**Authors:** Chen‐Ying Su, Mayur Jiyalal Prajapati, Jia‐Rou Lin, Cho‐Pei Jiang, Hsu‐Wei Fang

**Affiliations:** ^1^ Department of Chemical Engineering and Biotechnology National Taipei University of Technology Taipei Taiwan; ^2^ High‐Value Biomaterials Research and Commercialization Center National Taipei University of Technology Taipei Taiwan; ^3^ Department of Mechanical Engineering National Taipei University of Technology Taipei Taiwan; ^4^ Institute of Biomedical Engineering and Nanomedicine National Health Research Institutes Zhunan Town Taiwan; ^5^ Institute of Oral Tissue Engineering and Biomaterials National Yang Ming Chiao Tung University Taipei Taiwan

## Abstract

**Background:**

Absorbable barbed sutures have been widely used for minimally invasive facial rejuvenation. However, clinical lifting performance varies due to differences in suture geometry, which remain insufficiently quantified.

**Objective:**

The study aims to evaluate the effects of barb rotation angle, barb orientation, and pulling speed on the lifting performance and efficiency of the barbed sutures. The goal is to identify the optimized geometric design for facial lifting applications.

**Methods:**

The barbs were fabricated with four rotation angles (30°, 45°, 90°, and 180°) and four orientations (Forward, Reverse, Forward‐Reverse, and Reverse‐Forward). The fabricated sutures were tested at 10, 50, and 100 mm/min. Maximum lifting and holding displacements and lifting efficiency were quantified. Statistical analysis was conducted using one‐way ANOVA with Tukey's post hoc test (*p* < 0.05).

**Results:**

Higher pulling speed increased maximum lift but reduced efficiency due to slippage. The maximum lift was at 100 mm/min (2.03 ± 0.08 mm), whereas the highest lifting efficiency was at 10 mm/min (42.7%). For the barb rotation angles, the 90° configuration showed the highest lifting efficiency (35.5%) and superior anchor stability (*p* < 0.05). Regarding the barb orientation, the Forward orientation produced the largest lift (1.72 ± 0.18 mm), while the Forward‐Reverse orientation achieved the highest efficiency (35.5%). The optimized Forward‐Reverse 90° configuration exhibited improved holding capacity and reduced slippage compared to a commercial barbed suture.

**Conclusion:**

Barb geometry critically affects the lifting performance of the suture. The Forward‐Reverse 90° configuration shows the optimal lifting efficiency and stability, offering clinically relevant guidance for designing next‐generation facial lifting sutures.

## 1 Introduction

The demand for minimally invasive facial rejuvenation procedures continues to increase, as patients seek effective treatments with shorter recovery times and lower complication rates compared with traditional surgical facelift techniques [1, 2]. Thread lifting has therefore emerged as a widely adopted option for correcting facial ptosis, providing immediate repositioning of soft tissues while stimulating neocollagenesis for long term improvement [3, 4, 5, 6]. Absorbable barded sutures composed of materials such as polydioxanone (PDO), polylactic acid (PLA), and polycaprolactone (PCL) are frequently used because of their biocompatibility and predictable degradation profiles [7, 8]. Although clinical outcomes can be favorable, complications such as edema, bruising, thread migration, and the longevity of the lift vary considerably across studies [9, 10]. These inconsistencies highlight the need for a greater understanding of how the geometric design of barbed sutures affects anchoring performance and lifting stability.

The mechanical behavior of barbed sutures is strongly influenced by geometric parameters such as barb angle, spacing, depth, and directional arrangement. Prior investigations have demonstrated that barb geometry affects tissue engagement, pull‐out strength, and suture retention [11, 12, 13]. However, most published studies have evaluated commercially available sutures as complete products without isolating specific features such as barb rotation angle or directional orientation. This makes it difficult to determine the individual contribution of each geometric parameter to lifting efficiency or slippage resistance. Furthermore, clinical studies are often confounded by operator technique, anatomical variability, and subjective outcome assessment, limiting the reproducibility and generalizability of findings [10, 14]. These factors underscore that both suture design and material innovation play critical roles in optimizing thread lift outcomes [15, 16].

In vitro testing platforms using polydimethylsiloxane (PDMS) have gained increasing traction as reproducible and ethical alternatives to cadaveric or animal models. PDMS substrates have been shown to mimic soft‐tissue mechanical properties sufficiently for evaluating pull‐out strength and suture migration, while minimizing biological variability that complicates comparative analysis [17, 18]. Despite these advantages, prior PDMS‐based studies have not systematically combined controlled geometric fabrication of barbed sutures with a standardized mechanical lifting protocol.

Despite growing adoption, gaps remain in linking design parameters to clinical efficacy. Most clinical studies rely on subjective patient‐reported outcomes or surgeon assessments, with limited objective quantification of lifting force or retention [3, 9]. A clear research gap remains in describing the independent effects of barb rotation angle and directional orientation on the lifting performance, which have not been systematically quantified under controlled conditions. These parameters may significantly influence anchoring stability and displacement behavior, yet they have not been rigorously compared across a standardized experimental framework. Understanding these relationships is essential for evidence‐based optimization of thread design.

The present research hypothesizes that specific combinations of barb rotation angle and barb orientation will demonstrate superior lifting efficiency and anchoring stability compared with other configurations. To test this, sutures with different barb rotation angles (30°, 45°, 90°, and 180°), barb orientations (forward, reverse, forward‐reverse, and reverse‐forward), and test speeds (10, 50, and 100 mm/min) were fabricated and evaluated using a universal testing machine. The study quantifies two key outcomes: maximum lifting displacement and lifting efficiency. This research aims to (1) identify optimal barb geometries and orientations that maximize lifting efficiency, (2) establish a standardized in vitro framework for evaluating barbed sutures, and (3) provide quantitative data to guide clinical thread and improve patient outcomes.

By addressing these gaps, this study provides foundational biomechanical evidence to support the rational design of barbed sutures, potentially contributing to improved predictability and longevity of clinical thread‐lifting outcomes.

## 2 Design of Sutures

**FIGURE 1:**
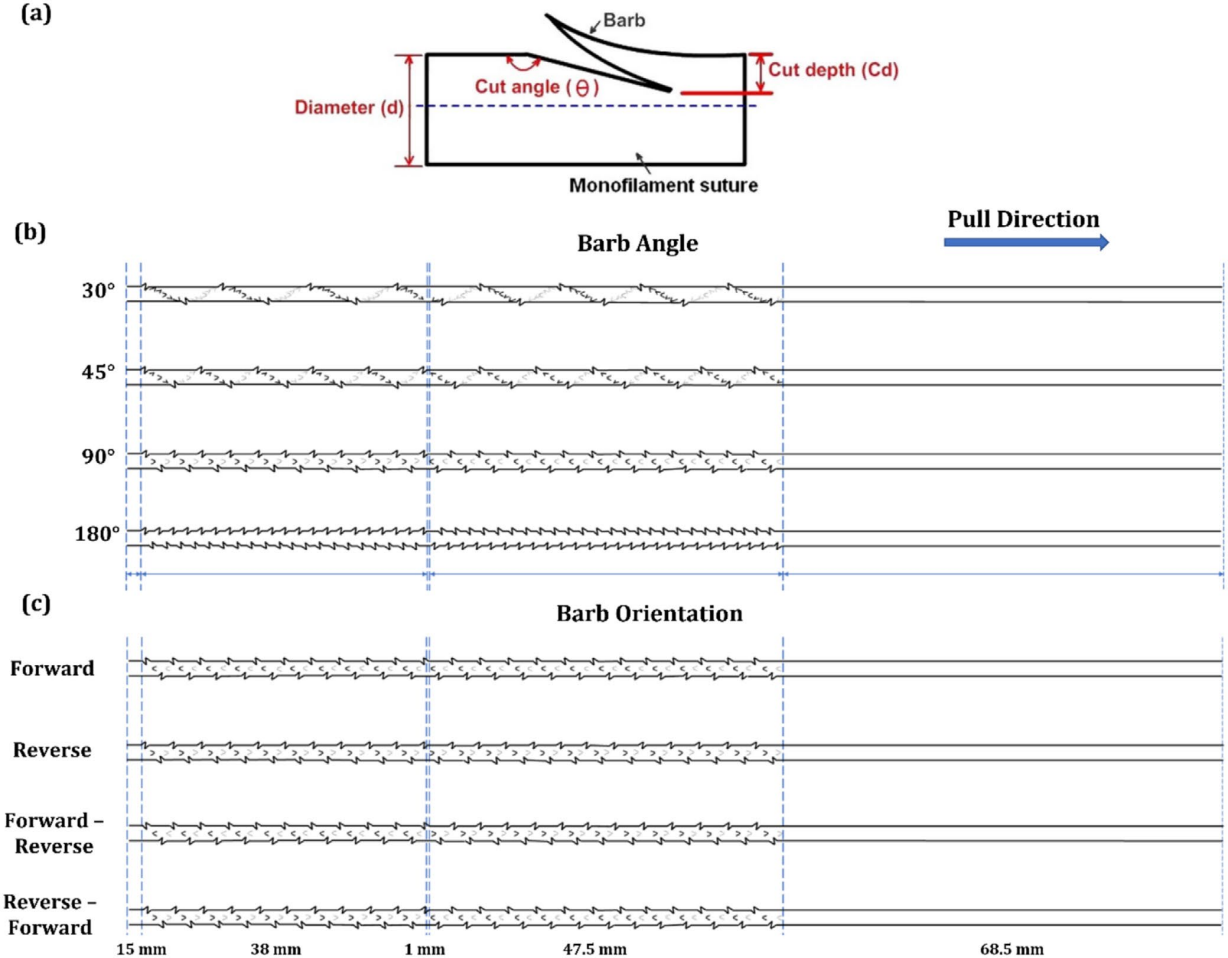
Design of barbed sutures (a) schematic representation of a barb, (b) barb angle, and (c) barb orientation.

The design of barbed sutures in this study concentrated on two critical geometric parameters: barb rotation and barb orientation. Figure 1a shows a schematic representation of a barb and its terminologies. These features determine the manner in which the suture engages with the surrounding soft tissue and, consequently, its lifting and retention capacity.

Barb rotation angle refers to the angular displacement between successive barbs around the circumference of the suture shaft. Four configurations were fabricated: 30°, 45°, 90°, and 180° (Figure 1b). A 30° rotation produced closely spaced barbs spiraling gradually along the suture, increasing the number of contact points with the substrate. A 45° rotation created moderately spaced barbs with balanced engagement and penetration. A 90° rotation resulted in barbs positioned orthogonally, maximizing surface contact per unit length. A 180° rotation placed barbs in opposite directions on alternating sides of the shaft, reducing density but potentially enhancing penetration depth at each barb site. By comparing these angles, the study aims to determine how barb spacing and circumferential distribution influence anchoring strength and lifting efficiency.

Four barb orientations based on the pull direction were designed to evaluate directional resistance (Figure 1c). In the Forward orientation, barbs are cut to resist displacement opposite to the insertion direction, anchoring primarily against withdrawal forces. In the Reverse orientation, barbs are cut to resist displacement opposite to the insertion direction, designed for stronger resistance to downward slippage. The bidirectional design features alternating forward and reverse barbs along the suture, which allows for anchoring in both directions. It has two configurations, Forward‐Reverse and Reverse‐Forward, based on the arrangements of the forward and reverse barbs along the pull direction.

## 3 Materials and Methods

### 3.1 Suture Fabrication

**FIGURE 2:**
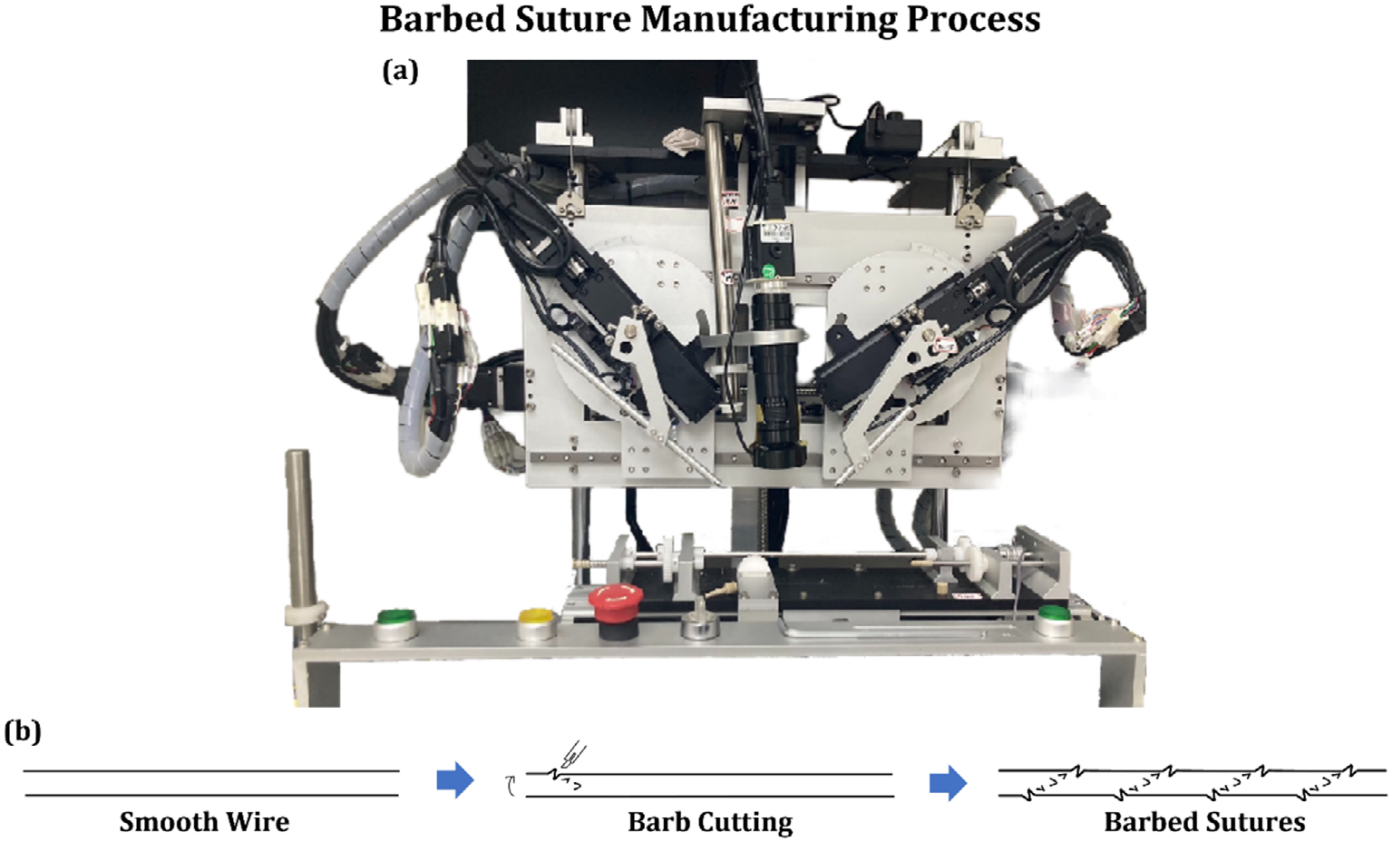
Barbed suture manufacturing process (a) manufacturing equipment and (b) suture manufacturing process.

Barbed sutures were fabricated using poly (L‐lactide‐co‐ɛ‐caprolactone) (P(LA/CL)) (Gunze Medical Division, Osaka, Japan) based monofilament suture material, which is an absorbable monofilament polymer with good tensile strength and biocompatibility. The fabrication was done using a self‐assembled suture cutting machine specifically developed for this purpose, as shown in Figure 2a. Prior to the operation, the machine axes, including the transverse, rotational, extrusion, and blade axes, were calibrated and returned to their home positions to ensure accuracy and consistency. The sutures were secured within the clamping system under slight tension to prevent displacement during processing. Figure 2b shows the barb generation process via the self‐assembled suture cutting machine.

Barb spacing was set to 0.95 mm with an intermediate spacing of 1 mm. The length of the suture was set to 170 mm and was divided into several functional zones to replicate realistic surgical application and control barb distribution. Each suture contained a total of 92 barbs distributed across the length, with the barbs arranged into two zones. The upper zone, which is the first zone in the direction of pull, contained 51 barbs distributed across the 47.5 mm zone. The lower zone contained 41 barbs distributed across the 38 mm zone (Figure 1). This zoning ensured that the barbs were not uniformly spread but instead reflected realistic thread‐lift sutures, where barb density may differ across the length of the suture to optimize anchoring and lifting performance during mechanical testing. For reproducibility, three independent sutures were prepared under each parameter condition. Following fabrication, all the machine axes were reset to their original positions to maintain instrument reliability for subsequent fabrications.

### 3.2 Substrate Fabrication

**FIGURE 3:**
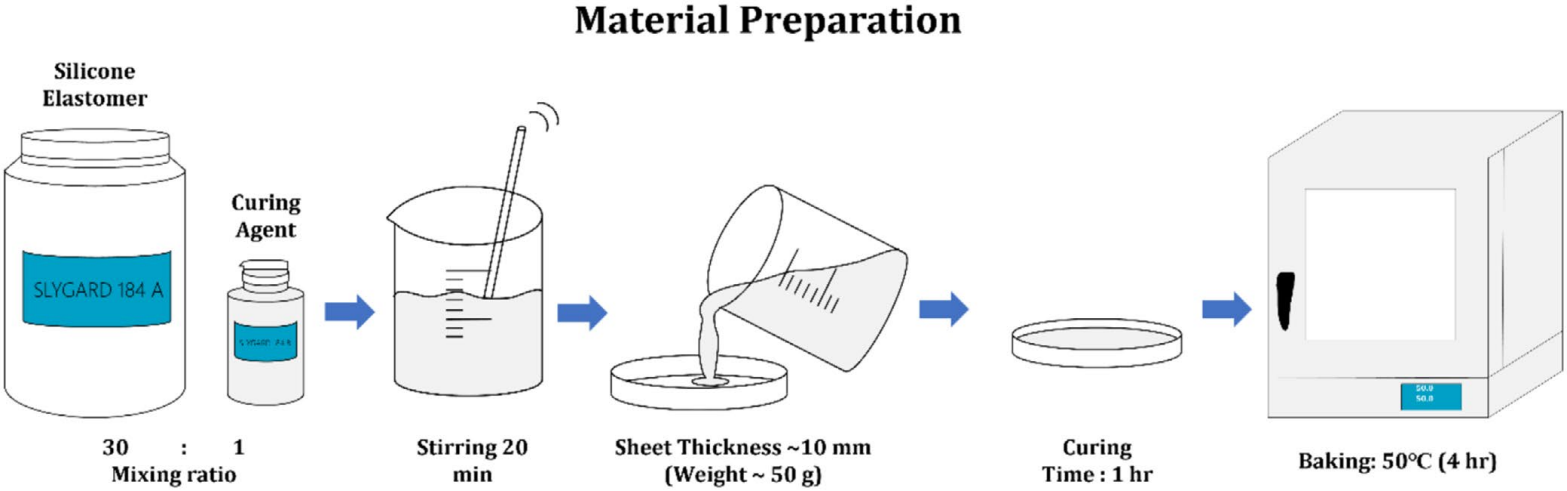
PDMS material preparation.

The polydimethylsiloxane (PDMS) substrates used for mechanical testing were prepared according to the manufacturer's instructions (SYLGARD 184, Dow Corning). As shown in Figure 3, the base polymer (SYLGARD 184A) and the curing agent (SYLGARD 184B) were mixed at a 30:1 weight ratio and continuously stirred for 20 min to ensure complete homogenization. The mixture was subsequently poured into culture dishes to yield substrates of ~10 mm thick, weighing ~50 g each. To minimize air bubbles, the mixture was left to stand for 1 h before curing. The prepared dishes were then placed in a drying oven (LDO‐9070A, Drawell Scientific, Chongqing, China) and cured at 50°C for 4 h to form solid PDMS substrates suitable for mechanical and lifting performance evaluation.

### 3.3 Hardness Testing

The hardness of the PDMS substrates was measured using a Shore Hardness Tester (LX‐AO, Wenzhou Sundoo Instruments Co. Ltd., China). Before testing, the device was calibrated and leveled to ensure accurate readings. Each PDMS specimen was positioned on the instrument platform, and measurements were obtained by vertically pressing the indenter into the substrate. To minimize spatial variation, five measurements were taken at positions located at least 15 mm from the edges and separated by a minimum of 6 mm, and the average value was reported as the hardness of the specimen. For validation of biomimetic similarity, fresh pork tissue samples, cut to approximately 50 × 50 mm^2^ from the subcutaneous to deep fat layers, were prepared and tested under the same conditions. The hardness values of the PDMS and pork tissues were then compared to confirm that the prepared PDMS substrates replicated the mechanical characteristics of the biological tissue with high fidelity.

### 3.4 Mechanical Testing

Mechanical testing of the sutures was performed on a Universal Testing Machine (UTM) (YM‐H3501‐A01, Taiwan). A load cell of 50 Newtons (N) was used to capture force responses. Crosshead speeds of 10, 50, and 100 mm/min were selected to examine the effect of strain rate on the barb anchoring and lifting performance. Each specimen was prepared by threading the barbed suture 25 mm through the PDMS substrate using an 18‐G blunt needle. The suture pass time, defined as the duration required to insert and correctly position the barbed suture beneath the substrate, was noted to ensure consistent handling conditions. The upper 20 mm of the suture was secured with adhesive tape to prevent premature slippage, and the free end was fixed within the upper clamp of the UTM. The PDMS substrate was positioned centrally within the lower fixture, secured by C‐clamps on the upper side and screws on the lower side to maintain stability during loading.

**FIGURE 4:**
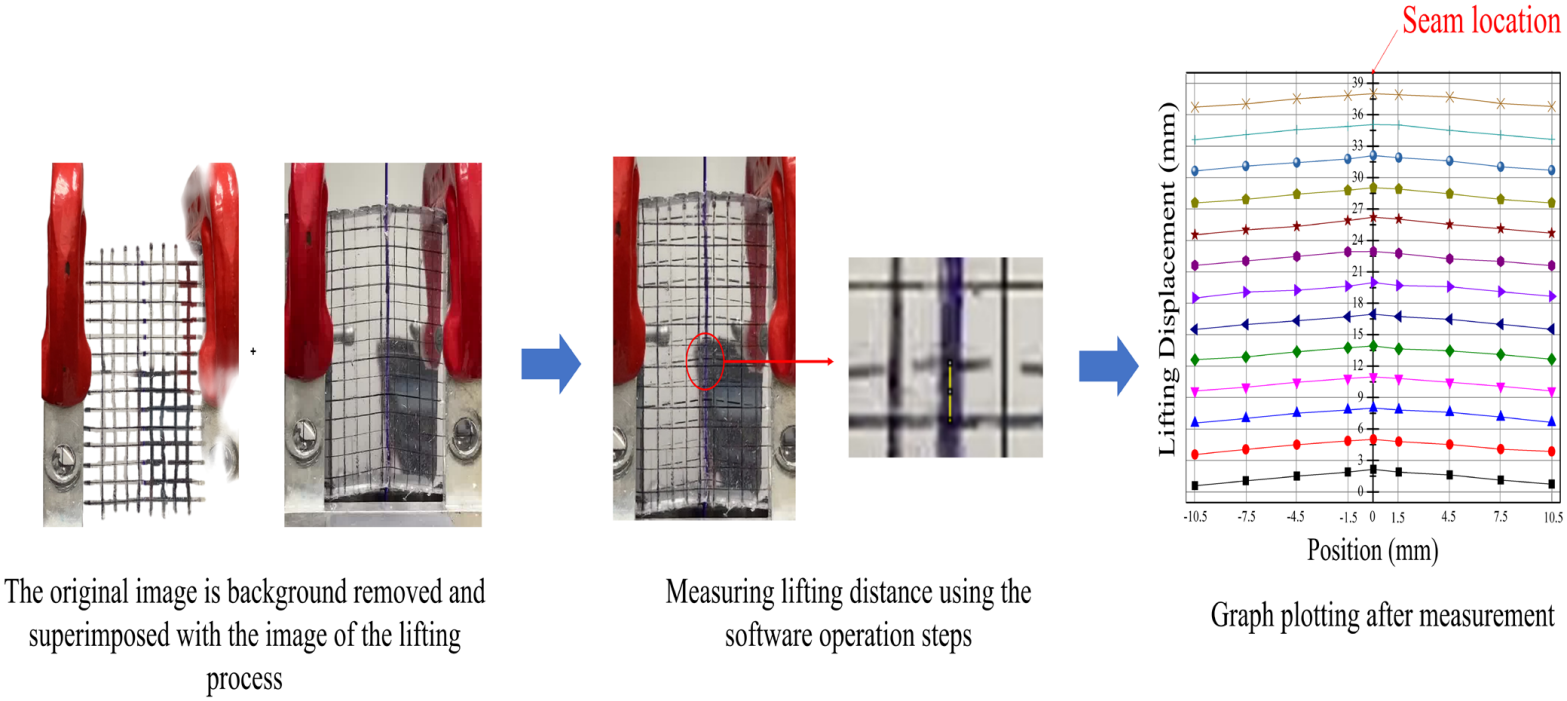
Experimental procedure to determine the maximum vertical lift of the PDMS.

Force‐displacement data was continuously recorded until barb slippage was observed. The maximum load (N) was defined as the peak force achieved before disengagement, while the displacement at maximum load (mm) was simultaneously measured. Video recordings of the trials were processed into image sequences and analyzed using ImageJ software to determine the maximum vertical displacement of the PDMS (mm), also known as the lifting displacement, which represents the initial vertical elevation achieved during tension (Figure 4). The retained deformation after unloading or over a defined period was regarded as the holding displacement, indicating the suture's ability to maintain the lift against relaxation or creep. Post‐peak monitoring allowed the determination of the residual lift (mm). Lifting efficiency was defined according to the relation:

$$\text{Lifting Efficiency}=\frac{\text{PDMS displacement}}{\text{Suture displacement}\;}\times 100$$

This standardized testing protocol provided reproducible measures of barb anchoring force, displacement, maximum lifting capacity, and efficiency across varying test speeds, rotational axis angles (30°, 45°, 90°, and 180°), and barb orientations (Forward, Reverse, Forward‐Reverse, and Reverse‐Forward).

### 3.5 Statistical Analysis

All the experiments were performed using three independently fabricated sutures per condition, with 13 positional measurements recorded for each suture. Data were expressed as mean ± standard deviation (SD). Differences among groups, including testing speeds, barb rotation angles, and barb orientations, were evaluated using one‐way analysis of variance (ANOVA). When ANOVA indicated a significant effect, pairwise comparisons were performed using Tukey's honestly significant difference (HSD) post hoc test. Statistical significance was defined as *p* < 0.05.

## 4 Results and Discussions

### 4.1 Hardness Testing

**FIGURE 5:**
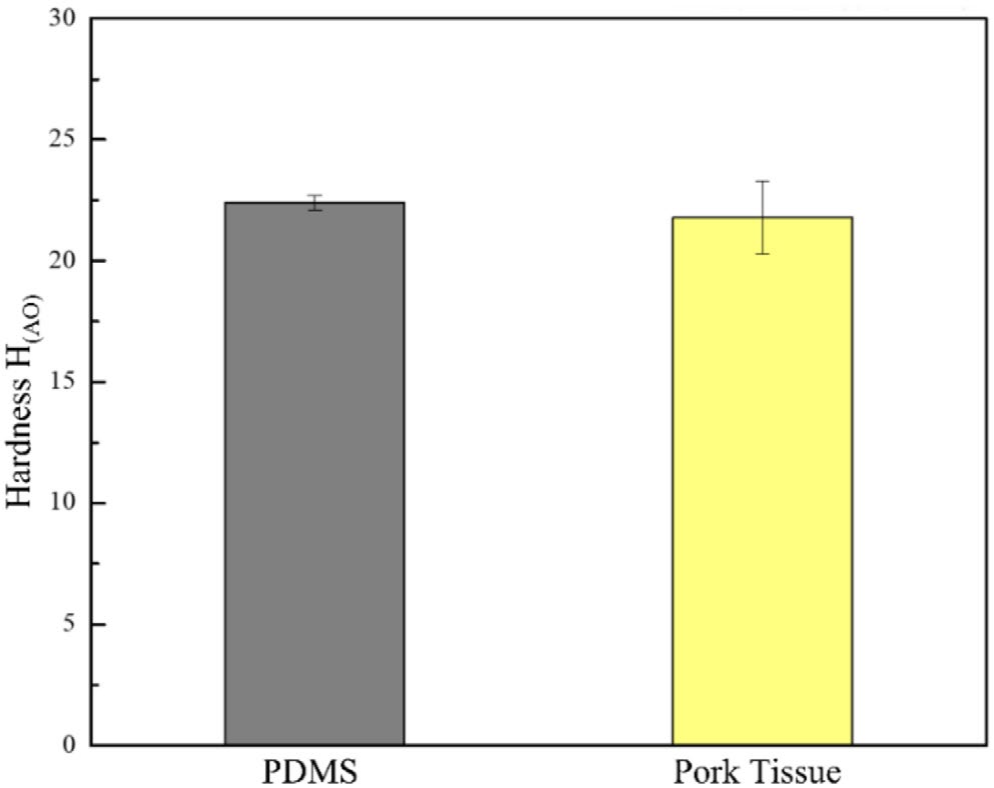
PDMS and pork tissue hardness test.

PDMS substrates exhibited a Shore AO hardness of 22.4 ± 0.29 H_(AO)_, while fresh porcine tissue taken from the subcutaneous to deep‐fat layers measured 21.8 ± 1.5 H_(AO)_ (Figure 5). PDMS is a suitable soft tissue analogue for subsequent lifting experiments. The close alignment in hardness is crucial since porcine tissue is often regarded as the gold standard for preclinical validation [19]. This strengthens the mechanical outcomes observed in PDMS as a reasonable approximation for the biological scenarios. Moreover, the low variability between specimens highlights the reproducibility of PDMS preparation, ensuring consistency across experiments. This step also prevents the misinterpretation of results, as performance differences can be attributed to suture design rather than inconsistencies in the substrate.

### 4.2 Effect of Test Speed on Lifting Performance

#### 4.2.1 Load—Displacement Behavior

**FIGURE 6:**
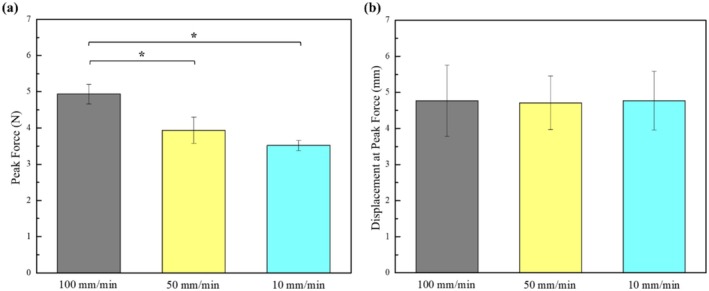
(a) Peak force vs. speed, and (b) displacement at peak force vs. speed (Data are presented as mean ± SD) *p < 0.05.

During the pulling test, the UTM machine pulls out the barbed wire, and the wire will grasp the PDMS initially, followed by a slip after it reaches the maximum peak load. Figure 6 and Table 1 display the peak load and its corresponding maximum displacement. From Figure 6a, it can be observed that 100 mm/min yields the maximum loading force, with a notable difference from the minimum force, which corresponds to 10 mm/min. This indicates that the faster the speed, the greater the generated force. Statistically, a significant difference was found between 100 mm/min and 50 mm/min, but 50 mm/min and 10 mm/min did not show any significant difference between them. From Figure 6b, it can be observed that there is no statistically significant difference in the displacements of the three speeds when they reach the peak load (*p* > 0.05), which means that the faster the speed, the greater the loading force per unit displacement.

**TABLE 1:** Peak force and displacement at the peak force (mean ± SD) at different crosshead speeds (statistical significance defined at *p* < 0.05).

Crosshead speed (mm/min)	Peak force (N)	Displacement at peak force (mm)
100	4.93 ± 0.27	4.81 ± 0.945
50	3.93 ± 0.36	4.77 ± 0.72
10	3.51 ± 0.13	4.79 ± 0.78

#### 4.2.2 Lifting Capacity and Efficiency

From Table 2 and Figure S1, it can be seen that the maximum lifting amount followed the load trend, with 100 mm/min yielding the largest lift and 10 mm/min the smallest, consistent with greater force producing more vertical displacement. 10 mm/min delivered the best lifting efficiency of 42.7%, whereas 100 mm/min produced the lowest lifting efficiency of 27.1%, indicating more slippage at higher rates despite larger absolute lift. Statistical analysis revealed that the average maximum lift differed significantly among the three speed conditions (*p* < 0.05). Tukey's HSD post hoc analysis showed that both 100 mm/min and 50 mm/min produced significantly higher lifting displacement compared with 10 mm/min. However, no statistically significant difference was observed between 100 mm/min and 50 mm/min, indicating comparable lifting performance at these two higher speeds. Together, these data show a trade‐off: faster pulls achieve higher peak lift but slip more, while slower pulls convert motion into sustained elevation more effectively.

**TABLE 2:** Effect of speed on maximum lift (mean ± SD), suture pass time, and lifting efficiency (statistical significance defined at *p* < 0.05).

Crosshead speed (mm/min)	Average maximum lift (mm)	Suture pass time (s)	Lifting efficiency (%)
100	2.03 ± 0.08	4.5	27.1
50	1.98 ± 0.08	7	34.0
10	1.85 ± 0.07	26	42.7

**FIGURE 7:**
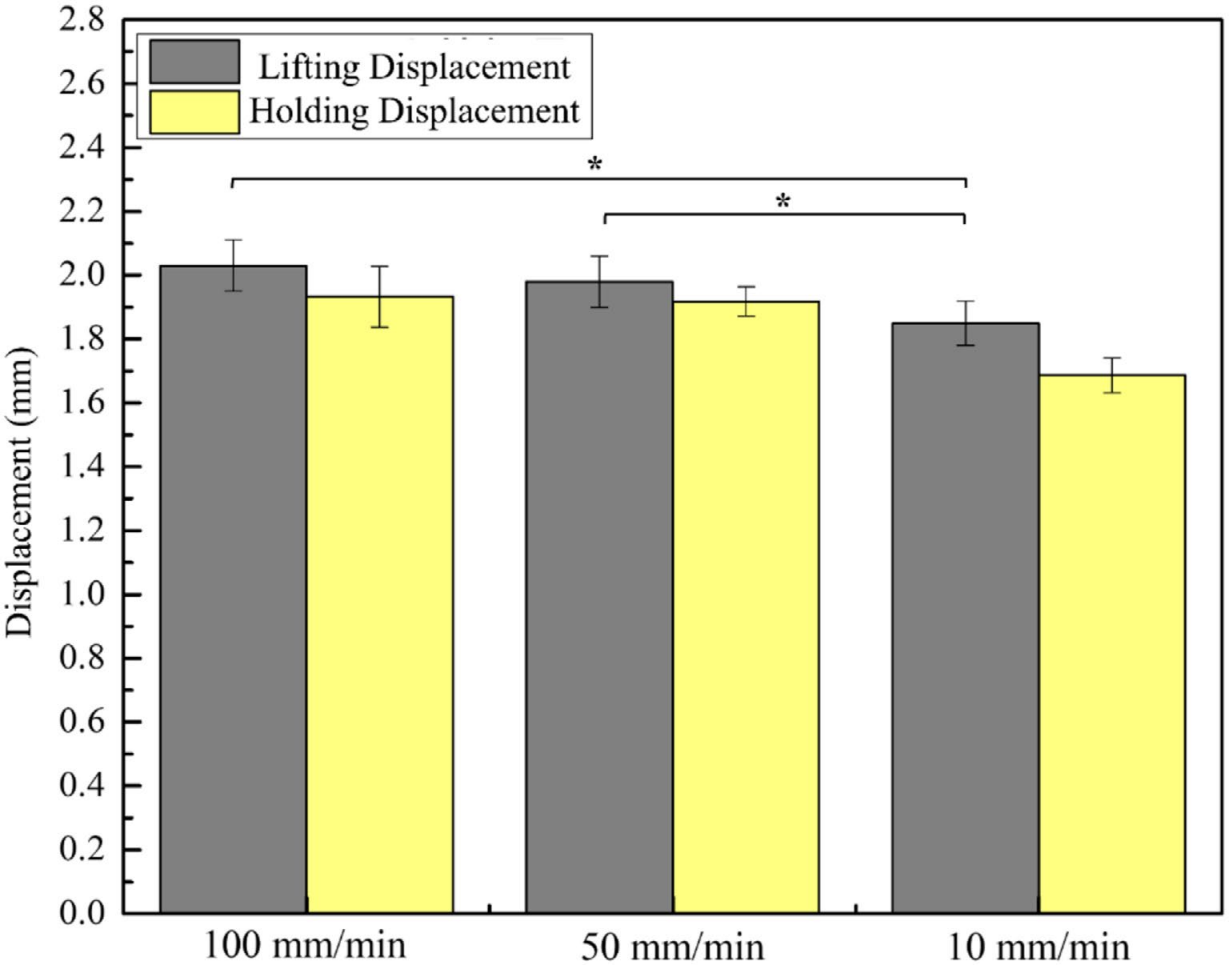
Comparison of avg. maximum displacement to avg. holding displacement for different speeds (data are presented as mean ± SD). *p < 0.05.

Figure 7 compares the average maximum lifting displacement with the subsequent hold displacement. Because the hooked seam will slip slightly after reaching its maximum lift, it decreases slightly. There is a significant difference in the lifting distance before and after 100 mm/min. As we know from the previous results, the faster the speed, the easier it is for the seam to slip. At 10 mm/min, there is a very significant difference before and after. This is because the speed is too slow and cannot support the pulled‐up PDMS, resulting in subsequent slipping. At 50 mm/min, the seam is relatively less likely to slip and can subsequently support the sliding PDMS.

### 4.3 Effect of Rotational Axis Angle on Lifting Performance

#### 4.3.1 Load—Displacement Behavior

**FIGURE 8:**
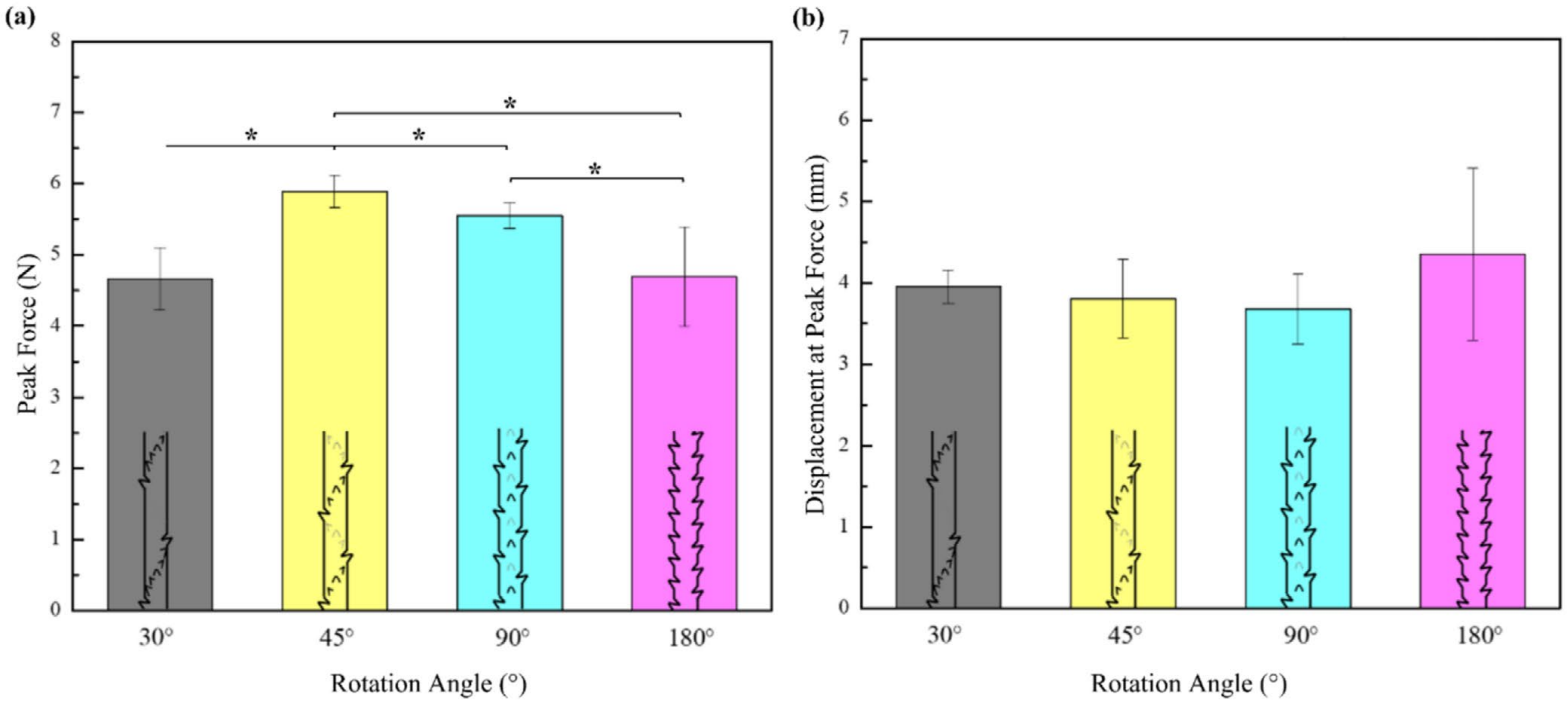
(a) Peak force vs. rotation angle, and (b) Displacement at peak force vs. rotation angle (data is presented as mean ± SD) *p < 0.05.

In Figure 8a and Table 3, it can be observed that the rotational axis angle of 45° has the maximum average loading force, while the 30° angle has the minimum loading force. In Figure 8b, minimum variation in the displacement at peak load can be observed with no statistically significant difference (*p* > 0.05). In the four rotation angles, the barb with 30° has 12 barbs in a circle, which resulted in the scattering of force, thus generating a smaller peak force, whereas the barbs with 45° and 90° rotation have 8 and 4 barbs, respectively. This resulted in a more concentrated force than a 30° rotation, and therefore, a higher peak force is generated. Barbs with 180° rotation have only 2 barbs in a circle, which results in force concentration on two sides, since the other sides are smooth. Thus, the resultant force is lower than that of the barb rotations of 45° and 90°, respectively. Statistically, there was a significant difference in peak force between 30° and 45°, 30° and 90°, 45° and 180°, and 90° and 180°, whereas 30° and 180°, and 45° and 90° do not show a statistically significant difference.

**TABLE 3:** Peak force and displacement at the peak force (mean ± SD) at different barb rotations (statistical significance defined at *p* < 0.05).

Rotation angle	Peak force (N)	Displacement at peak force (mm)
30°	4.67 ± 0.45	3.92 ± 0.22
45°	5.89 ± 0.22	3.86 ± 0.55
90°	5.55 ± 0.17	3.74 ± 0.46
180°	4.79 ± 0.69	4.41 ± 0.86

#### 4.3.2 Lifting Capacity and Efficiency

The lifting capacity and efficiency are depicted in Table 4 and shown in Figure S2. 90° shows the maximum average lift, whereas 30° shows the minimum lift, which was slightly different from the force trend shown in Figure 8a. The force at a 45° rotation is greater than that at 90°, but the lift was lower. This was because after reaching the highest point during the ascent, the suture slipped slightly, losing its secure anchoring position in the tissue. This caused the PDMS to slide slightly, resulting in greater resistance to the suture. Consequently, the load force was higher, but the lift was lower. Statistically, the effect of the rotation angle on the maximum lifting capacity was significant (*p* < 0.05). All the pairwise comparisons exceeded the HSD threshold. Among these, the 90° configuration exhibited the highest lifting displacement, while the 30° configuration showed the worst performance, confirming the critical role of the rotational angle in the lifting efficiency.

**TABLE 4:** Effect of rotation angle on maximum lift (mean ± SD), suture pass time, and lifting efficiency (statistical significance defined at *p* < 0.05).

Rotation angle	Average maximum lift (mm)	Pass time (s)	Lifting efficiency (%)
30°	0.81 ± 0.10	1.5	32.4
45°	1.21 ± 0.10	2	33.8
90°	1.48 ± 0.04	2.5	35.5
180°	1.00 ± 0.06	2.5	24

Regarding lifting efficiency, the barbed suture at a 90° rotation has the highest efficiency of 35.5%, while the barbed suture at a 180° rotation axis has the lowest efficiency of 24.0%. This is because the 180° rotation has bars only on two sides, with the remaining surfaces being smooth, resulting in poorer anchoring than the other three.

**FIGURE 9:**
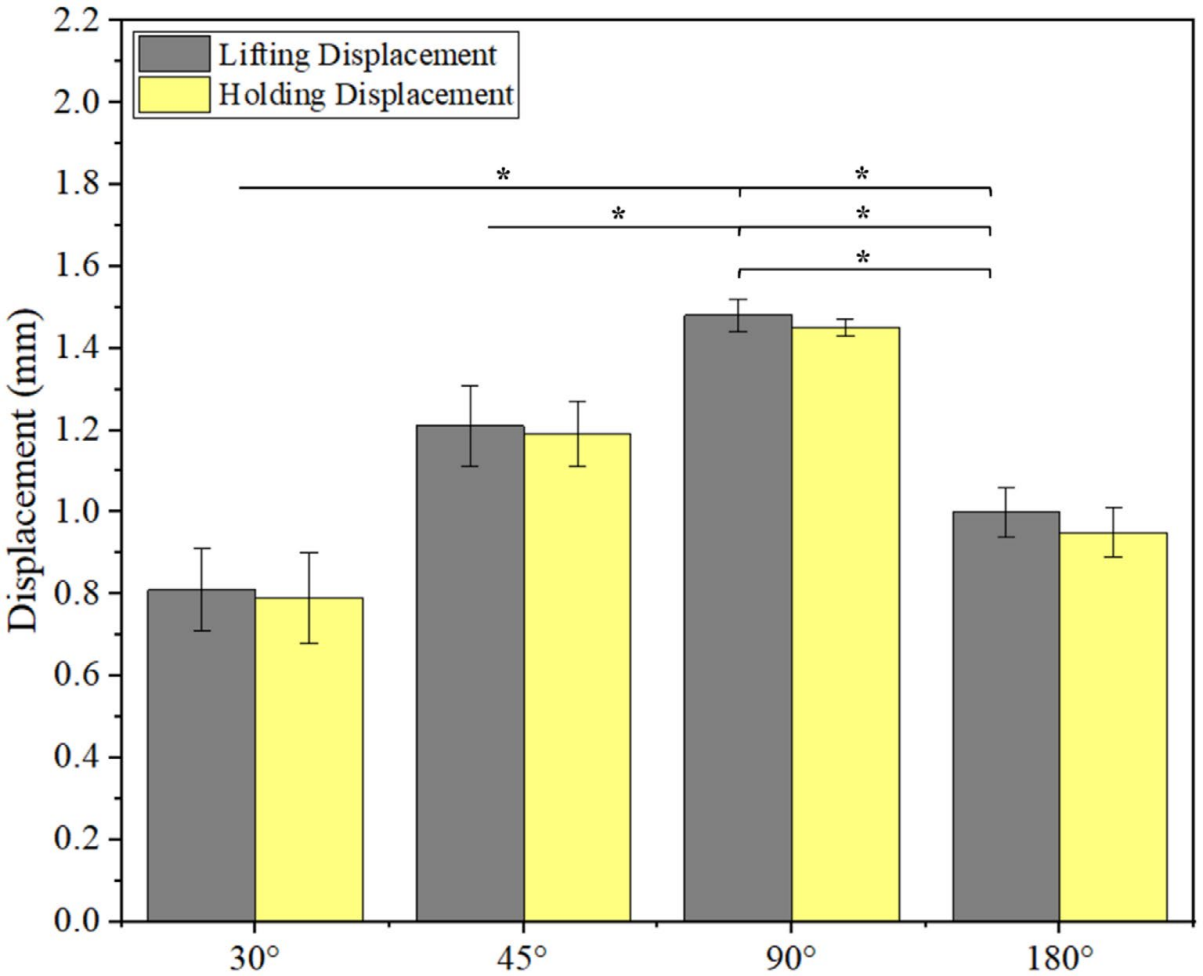
Comparison of avg. maximum displacement to avg. holding displacement for different barb rotations (data is presented as mean ± SD) *p < 0.05.

Figure 9 shows the lifting and the holding displacements for all the barb rotations. The 180° rotation demonstrates the largest difference between the lifting and holding displacements. The barbs are only on the two sides, so the smooth surface can't support the PDMS after rising. At other angles, the barbs are scattered on all sides, so the PDMS is likely to slide down after rising.

### 4.4 Effect of Barb Orientation on Lifting Performance

#### 4.4.1 Load—Displacement Behavior

**FIGURE 10:**
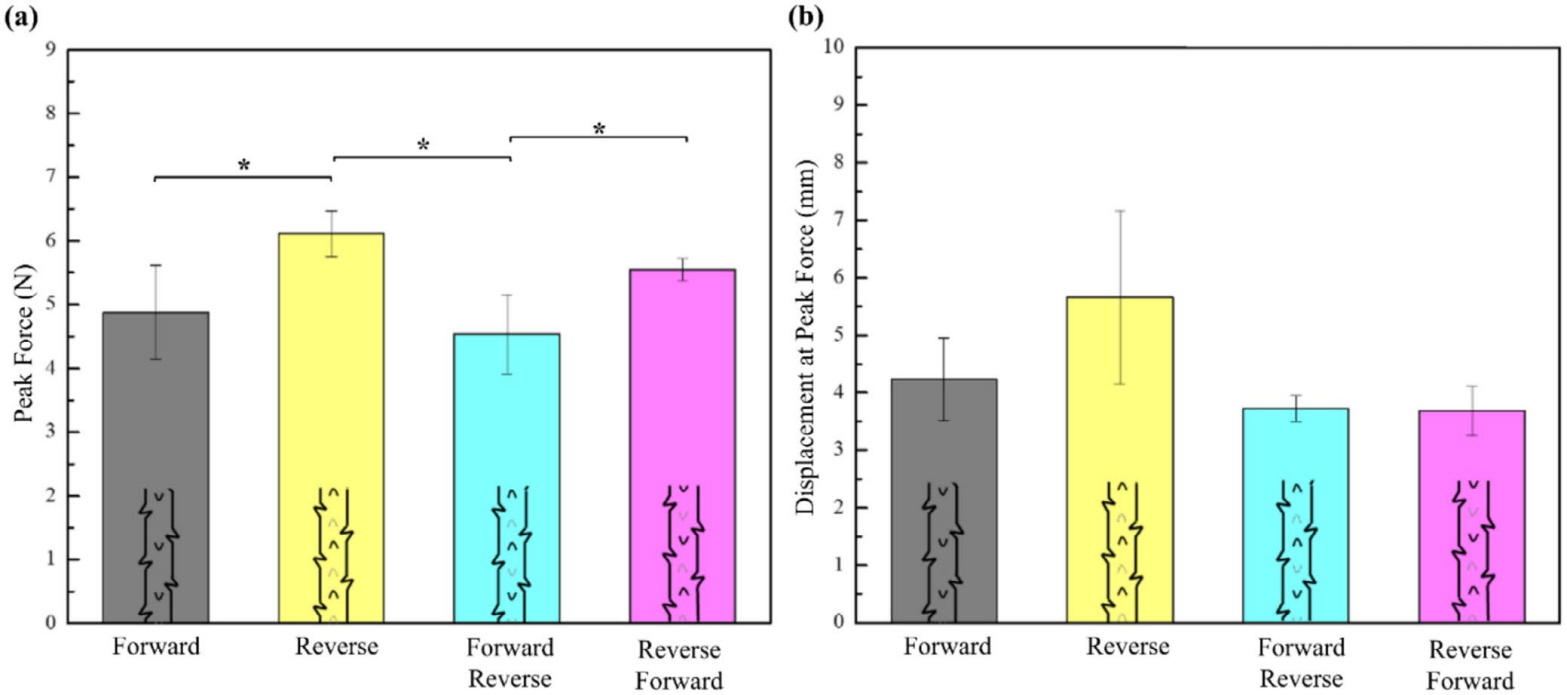
(a) Peak force vs. barb orientations, and (b) Displacement at peak force vs. barb orientations (data is presented as mean ± SD) *p < 0.05.

Figure 10 and Table 5 show the peak load and corresponding displacement performance of barbed sutures with different orientations. From Figure 10a, it can be seen that the reverse direction has the highest peak load, while Forward‐Reverse has the lowest peak loading performance. Furthermore, both the Forward and Reverse directions show lower loading performances than the Reverse direction. Looking at the displacement graph in Figure 10b, it can be seen that the displacement at maximum load is greater for the Reverse direction as compared to the other configurations. The Reverse direction barb has the highest load because the reverse barb penetrates the PDMS when pulled, which results in a smaller force‐bearing area at the tip of the barb. Consequently, the pressure is greater, and the load increases. Also, the higher displacement at the peak load for Reverse orientation indicates that while the load is higher, the force generated per unit is not high. A statistically significant difference was observed in the Tukey post hoc test between the Forward and Reverse, Reverse and Forward‐Reverse, and Forward‐Reverse and Reverse‐Forward orientations.

**TABLE 5:** Peak force and displacement at the peak force (mean ± SD) at different barb orientations (statistical significance defined at *p* < 0.05).

Barb orientation	Peak force (N)	Displacement at peak force (mm)
Forward	4.89 ± 0.75	4.42 ± 0.89
Reverse	6.29 ± 0.25	5.87 ± 1.21
Forward—Reverse	4.54 ± 0.12	3.89 ± 0.30
Reverse—Forward	5.65 ± 0.61	3.77 ± 0.41

#### 4.4.2 Lifting Efficiency

The lifting performance of the barbed sutures with respect to different orientations is shown in Figure S3 and tabulated in Table 6. It can be seen that the Forward orientation exhibits the highest maximum lift, while the Forward‐Reverse shows the least. This is because the forward barbs push the tissue forward, causing less damage to surrounding structures. Thus, as long as the suture continues to pull, it will lift upward until reaching its limit. Although reverse barbed sutures anchor by piercing the PDMS to pull upward, they cause greater tissue disruption, resulting in poorer overall elevation. In Reverse‐Forward orientation, the Reverse orientation, which is first in the direction of pull, pierces the tissue that leads to tissue disruption in the surroundings. While the Forward orientation followed by the Reverse does provide upward lift, the tissue slippage as a result of the disruption results in lower overall lift. Conversely, the Forward‐Reverse configuration works differently; the forward part pulls the tissue upwards. The Reverse orientation, followed by the Forward, encounters a greater resistance from tissue due to the initial lift. This lowers the force that could damage the tissue. Thus, overall lift is superior to that of the Reverse‐Forward orientation. The barb orientation also showed a significant effect on the lifting performance (*p* < 0.05). Post hoc analysis revealed that the Forward orientation produced significantly greater lifting displacement compared to the other configurations. The Reverse‐Forward orientation showed the lowest lifting capacity and differed significantly from all other orientations. No statistically significant difference was observed between the Reverse and Reverse‐Forward orientations.

**TABLE 6:** Effect of barb orientation on maximum lift (mean ± SD), suture pass time, and lifting efficiency (statistical significance defined at *p* < 0.05).

Barb orientation	Average maximum lift (mm)	Suture pass time (s)	Lifting efficiency (%)
Forward	1.72 ± 0.18	5 s	20.2%
Reverse	1.53 ± 0.12	5.5 s	16.0%
Reverse—Forward	1.33 ± 0.07	5.5 s	15.0%
Forward—Reverse	1.48 ± 0.04	2.5 s	35.5%

Regarding the lift efficiency, the Forward‐Reverse orientation offered the highest efficiency of 35.5%, while the Reverse‐Forward orientation offered the least efficiency of just 15%. Although Forward and Reverse sutures yield higher lifting volumes, they take longer to reach maximum lifting capacity, resulting in lower overall efficiency.

**FIGURE 11:**
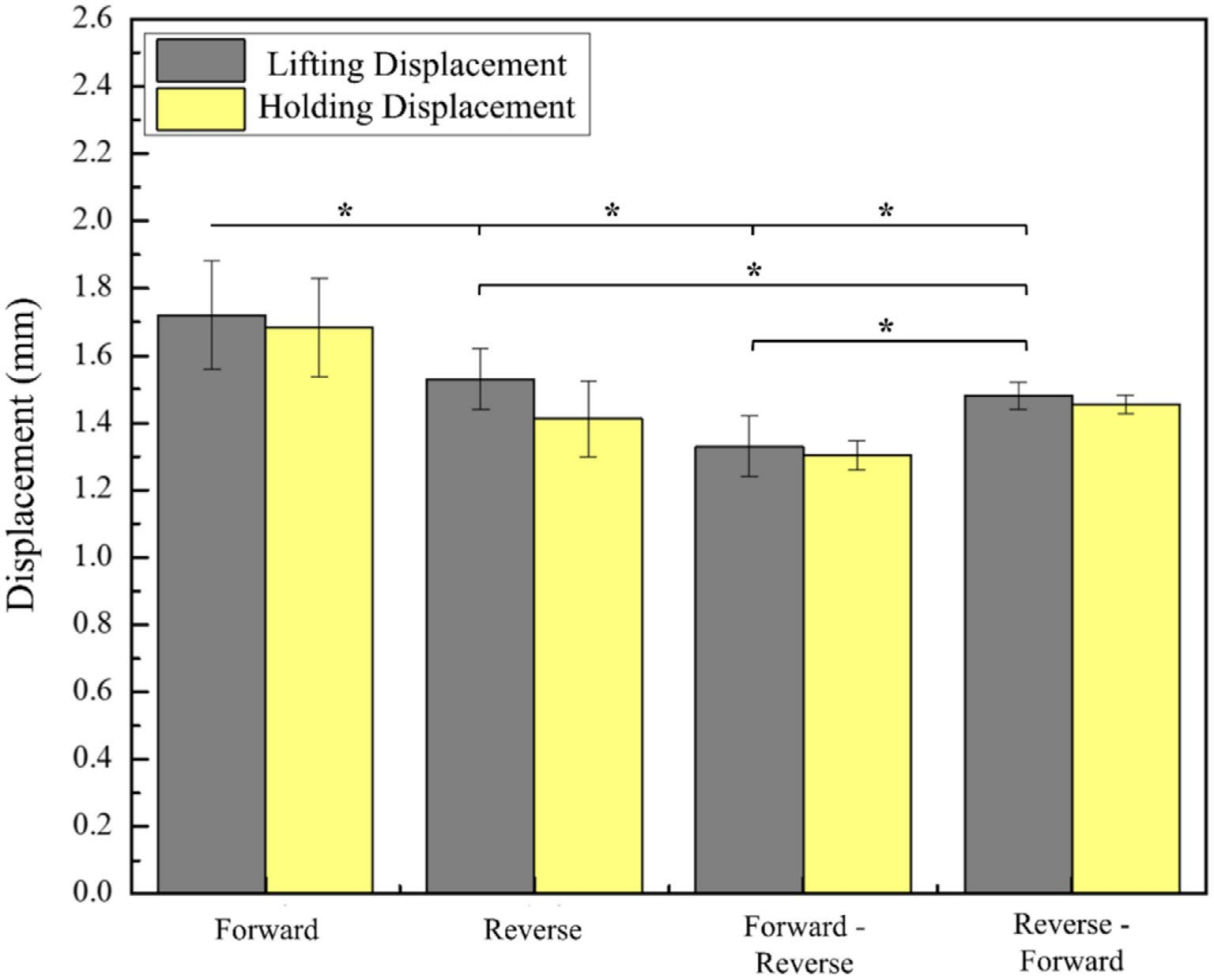
Comparison of the average maximum displacement to the average. Holding displacement for different barb orientations (data is presented as mean ± SD) *p < 0.05.

As can be seen in Figure 11, only the Reverse shows significant slippage compared to other orientations. This is because after the reverse barbs penetrate the PDMS, they anchor and pull the PDMS up, causing localized damage over time. This causes the PDMS to slide down more compared to other orientations.

### 4.5 Comparison With Prior Studies

The present findings are consistent with the previous studies showing that the barb geometry critically governs the lifting behavior and the efficiency of the barbed sutures [11, 12, 13]. Table 7 shows the comparison of the present study with the prior literature. Earlier porcine and cadaveric tissue cases demonstrated that the reverse‐oriented barbs showed the highest peak force as a result of a deeper tissue penetration, but cause localized tissue damage and post‐peak slippage [12, 18]. This behavior aligns with the observation of the Reverse barbs in the current study that showed a higher peak load but lower lifting efficiency and the corresponding holding displacement. In contrast, bidirectional barb designs have been clinically favored for balanced anchoring and lifting behavior [3, 8]. The present study provides quantitative biomechanical evidence supporting this preference, showing that the Forward‐reverse configuration achieved the best lifting efficiency with improved holding stability. Importantly, unlike the previous studies that evaluated commercial sutures as integrated products, the present study isolates geometric features such as orientation and rotation angle under standardized conditions, showing that the 90° barb rotation provides superior circumferential stress distribution and anchoring efficiency. These findings extend existing literature by offering mechanistic insight into how specific geometric parameters affect the lifting performance.

**TABLE 7:** Comparison of the present study with the prior literature.

Study	Model/tissue	Design variables studied	Key findings	Limitations
Ingle and King [11]	Ex vivo skin and tendon tissue	Barb geometry, pull‐out strength	Demonstrated that barb size and spacing strongly influence the pull‐out behavior and anchoring force	Did not isolate the circumferential barb rotation. The lifting displacement and efficiency were not evaluated
Sasaki et al. [12]	Human Cadaver	Barb's orientation, holding, and slippage	Reverse‐oriented barbs produced higher peak holding forces but exhibited greater post‐peak slippage	No controlled comparison of individual geometric parameters
Zaruby et al. [13]	In vivo animal model	Barbed vs. conventional sutures	Barbed sutures improved holding strength compared to the smooth sutures, and altered tissue‐suture interaction	Focused on wound closure rather than lifting mechanics. Geometrical parameters were not varied
Hong et al. [18]	PDMS and porcine tissue	Pull‐out force, migration resistance	PDMS provided reproducible results comparable to porcine tissue for evaluating suture anchoring	Did not evaluate lifting displacement or efficiency. Limited geometric parameters were varied
Present study	PDMS	Barb rotation angle, orientation, pull speed	Identified Forward‐Reverse orientation with 90° configuration, optimal for lifting efficiency and holding stability	In vitro model may not capture biological remodeling or long‐term clinical response

### 4.6 Comparison With Commercially Available Products

**FIGURE 12:**
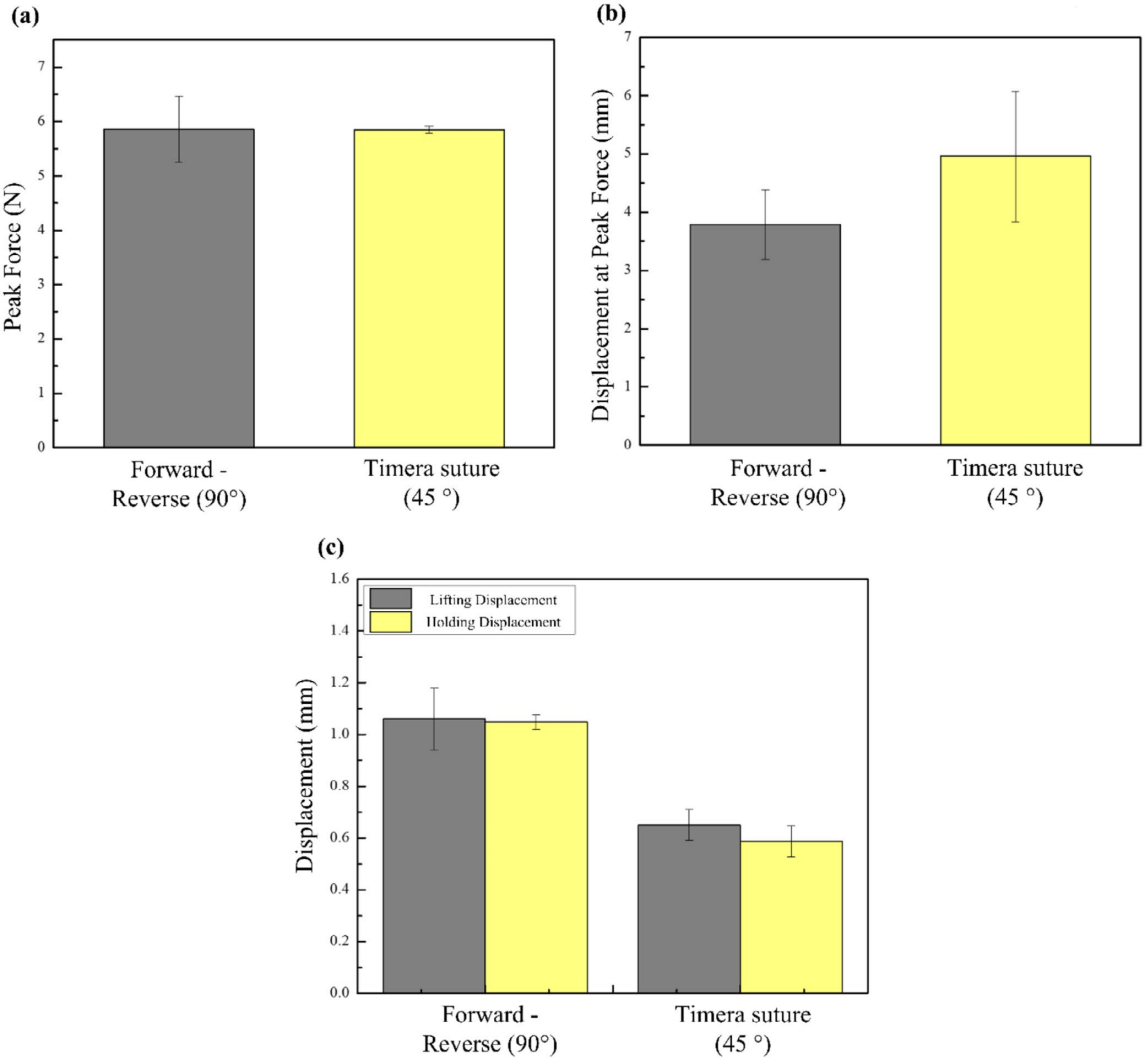
(a) Peak force, (b) Displacement at peak force, and (c) Avg maximum displacement to avg. holding displacement comparisons for Forward—Reverse 90° and Miracle suture.

The above experimental analysis has shown that the optimal suture design is a 90° barb rotation with a Forward‐Reverse barb orientation. To validate its efficacy, it was compared with a commercially available suture, Miracle Thread Knotless Tissue‐Closure Device (Diamond Biotechnology Co. Ltd.; Taipei, Taiwan), because its barb design served as a reference during the initial designing phase [20]. The Miracle suture features a 45° barb rotation with a Forward‐Reverse barb orientation. The average peak force and the maximum displacement at the peak force are shown in Figure 12a,b, respectively. There is no significant difference between the two in terms of average peak force, but the maximum displacement at the peak force is higher in the Miracle suture when compared to the Forward‐Reverse 90°. However, the holding capacity, as shown in Figure 12c, reveals that the Timera suture exhibited lower lifting capacity than the Forward‐Reverse 90°. This result aligns with the previous experiments involving different barb rotations. Also, the slipping in Miracle is much more pronounced than in Forward‐Reverse 90°. Nevertheless, since Miracle reached its maximum lift within 2 s, the lifting efficiency comes out to be 19.5%, which is slightly better than the lifting efficiency designed for the experiments of this study.

### 4.7 Clinical Implications of Barb Design

The mechanical lifting behavior observed provides important insights into how barbed suture design may influence tissue response and clinical performance. Higher maximum lift reflects the ability of the suture to achieve immediate soft‐tissue elevation, a key determinant of early aesthetic outcome. However, increased slippage at higher pull speeds and certain geometric configurations suggests reduced anchoring stability, which clinically may manifest as early loss of lift. Conversely, higher lifting efficiency and holding displacement indicate more effective load transfer from the suture to the surrounding tissue. This potentially reduces localized tissue damage and improves the durability of the lift.

The superior performance of the 90° barb orientation can be attributed to a more uniform circumferential distribution of barbs, which likely reduces stress concentration at individual contact points and promotes balanced tissue engagement. Clinically, such stress distribution may lower the risk of localized tissue tearing and inflammation. Similarly, the Forward‐Reverse orientation demonstrated optimal lifting efficiency by combining effective upward traction with reduced tissue disruption due to thread penetration. This balance may translate to improved procedural control, shorter operative time, and postoperative discomfort.

From a clinical perspective, the Forward‐Reverse 90° configuration offers a favorable compromise between immediate lifting capacity and long‐term anchoring stability. These mechanical characteristics are consistent with desirable clinical responses, including sustained tissue repositioning, reduced slippage, and predictable performance across different pulling speeds. Although the PDMS substrate used in this work demonstrated similar hardness to that of the porcine tissue and offered excellent reproducibility, it does not fully replicate the complex mechanical and biological features of the human facial tissue. Actual human tissues show heterogeneous composition, anisotropic viscoelastic behavior, and time‐dependent remodeling behavior that cannot be captured by a homogeneous synthetic model. In addition, PDMS does not account for the biological phenomena such as inflammation, fibrosis, collagen induction, or vascularity, all of which may influence long‐term clinical outcomes following thread lifting. Therefore, the results of the present study should be interpreted as controlled in vitro biomechanical behavior that shows the effect of suture design rather than direct predictors of in vivo performance.

While the present study used an in vitro PDMS model, the observed trends provide a biomechanical rationale that may guide the design of the next‐generation absorbable barbed sutures and inform evidence‐based selection of the thread geometry in facial rejuvenation. Future studies, incorporating ex vivo human or animal tissue models and clinical validation, will be necessary to further translate these findings into clinical practice. These investigations are further required to evaluate the biological phenomenon, such as inflammation, fibrosis, collagen induction, and long‐term anchoring performance, which cannot be captured by the synthetic substrates. Also, comparison with other commercial threads could provide direct evidence of clinical efficacy, durability of lift, and patient‐reported responses. Incorporating image‐based assessments and long‐term follow‐up would further strengthen translational relevance and support evidence‐based optimization of facial lifting sutures.

## 5 Conclusion

This study systematically examined the influence of the barb's geometrical features, such as the rotation angle and the orientation, on the lifting and anchoring behavior of the barbed sutures using a controlled in vitro model. The results show that the suture geometry plays a crucial role in balancing the lifting capacity, anchoring stability, and resistance to post‐peak slippage. In particular, a uniform circumferential distribution of the barbs combined with a bidirectional orientation was shown to promote stable load transfer and efficient tissue engagement.

From a translational perspective, the results indicate that by optimizing barb geometry, the lifting efficiency can be improved while minimizing tissue disruption. The identified Forward‐reverse with 90° configuration (35% lifting efficiency) offers a favorable balance between lift and anchoring performance, which are desirable characteristics for predictable and durable clinical outcomes.

Although derived from an in vitro platform, these findings provide a biomechanical rationale for the optimal design of next‐generation absorbable barbed sutures. By linking the geometric parameters to the functional lifting behavior, this work offers a practical guide for device development and optimization and may assist clinicians and manufacturers in selecting or designing thread configurations that enhance procedural reliability.

## Funding

This work was supported by the Ministry of Economic Affairs, Taiwan, 112‐EC‐17‐A‐19‐S6‐025.

## Ethics Statement

The authors have nothing to report.

## Consent

The authors have nothing to report.

## Conflicts of Interest

The authors declare no conflicts of interest.

## Supporting information


**Figure S1:** Avg. maximum vertical lift at different positions across the PDMS at (a) 100 mm/min, (b) 50 mm/min, and (c) 10 mm/min speeds.
**Figure S2:** Avg. maximum vertical lift at different positions across the PDMS in (a) 30°, (b) 45°, (c) 90°, and (d) 180° rotation angles.
**Figure S3:** Avg. maximum vertical lift at different positions across the PDMS in (a) Forward, (b) Reverse, (c) Forward—Reverse and (d) Reverse—Forward barb orientations.

## Data Availability

The data that support the findings of this study are available from the corresponding author upon reasonable request.
