# Supplementary material for: Lifting Efficiency of Barbed Sutures for Potential Face Lifting Application: A Parametric Analysis
Source: J Cosmet Dermatol. 2026 Mar 6;25(3):e70730. doi: 10.1111/jocd.70730 (PMC12965929; doi:10.1111/jocd.70730)
Supplement: Supplementary file 1 — Figure S1: Avg. maximum vertical lift at different positions across the PDMS at (a) 100 mm/min, (b) 50 mm/min, and (c) 10 mm/min speeds. Figure S2: Avg. maximum vertical lift at different positions across the PDMS in (a) 30°, (b) 45°, (c) 90°, and (d) 180° rotation angles. Figure S3: Avg. maximum vertical lift at different positions across the PDMS in (a) Forward, (b) Reverse, (c) Forward—Reverse and (d) Reverse—Forward barb orientations. [file JOCD-25-e70730-s001.docx]

Supplementary Information


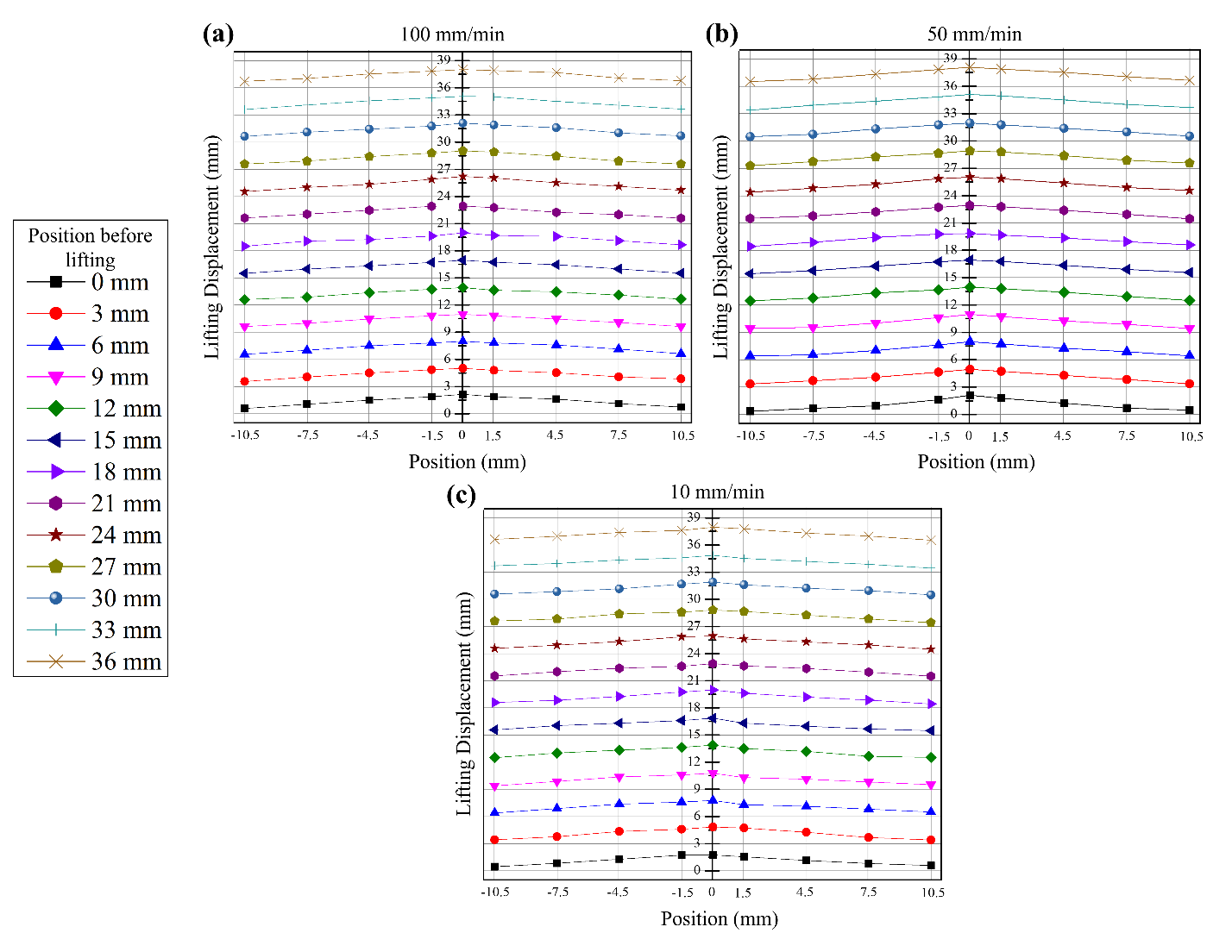


Figure S1: Avg. maximum vertical lift at different positions across the PDMS at (a) 100 mm/min, (b) 50 mm/min, and (c) 10 mm/min speeds


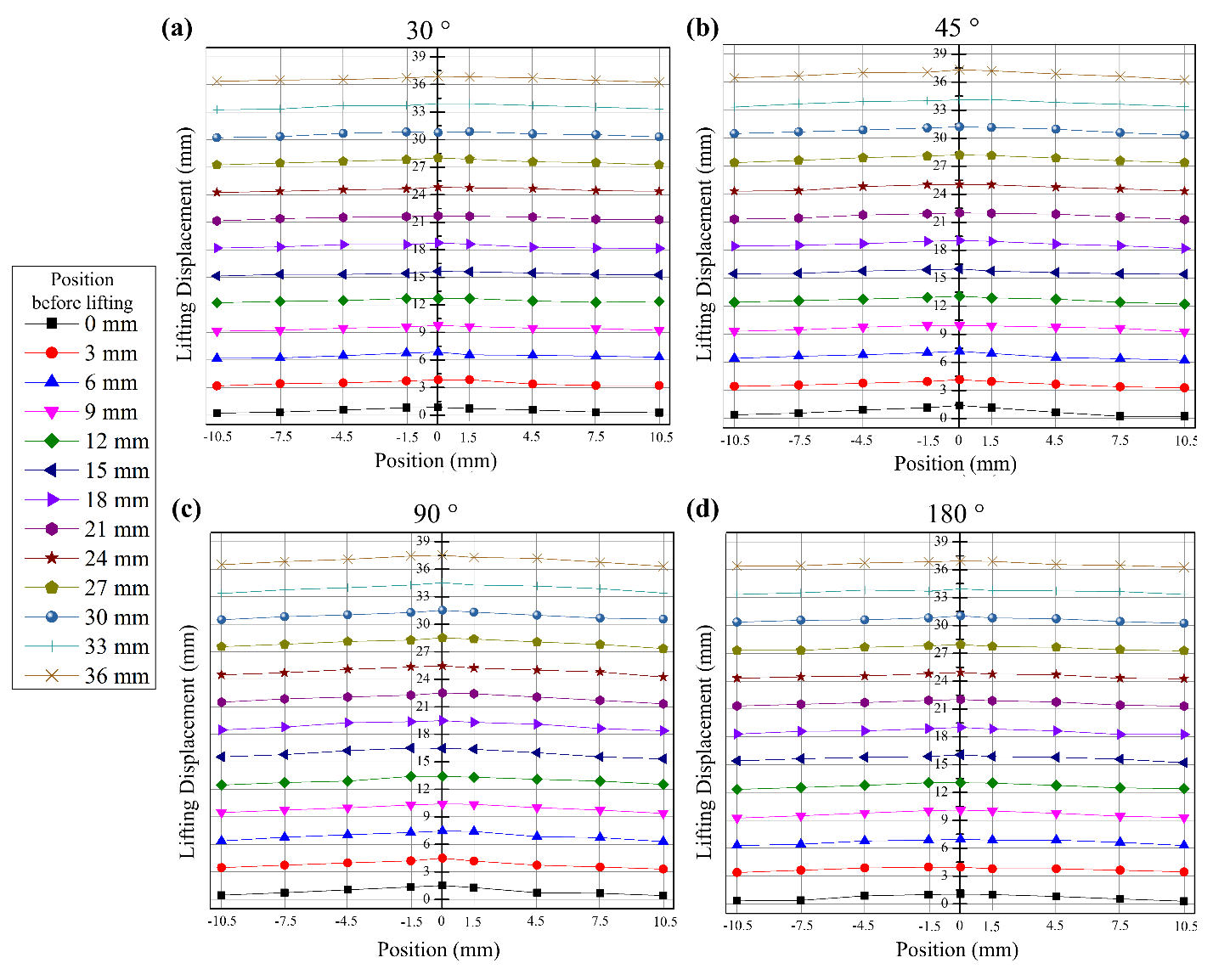


Figure S2: Avg. maximum vertical lift at different positions across the PDMS in (a) 30°, (b) 45°, (c) 90° and (d) 180° rotation angles


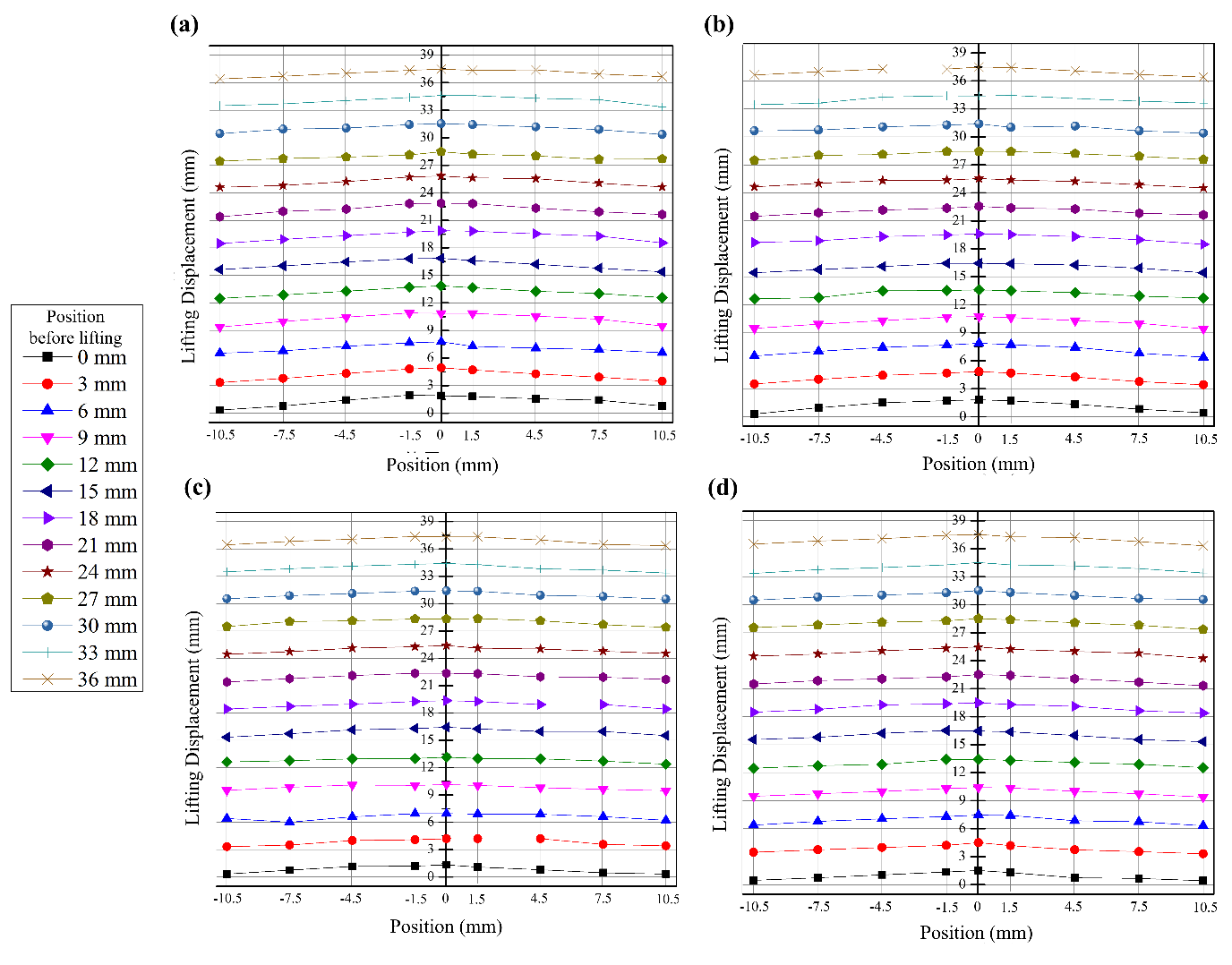


Figure S3: Avg. maximum vertical lift at different positions across the PDMS in (a) Forward, (b) Reverse, (c) Forward – Reverse and (d) Reverse – Forward barb orientations
